# Comparison of high and low intensity contact between secondary and primary care to detect people at ultra-high risk for psychosis: study protocol for a theory-based, cluster randomized controlled trial

**DOI:** 10.1186/1745-6215-14-222

**Published:** 2013-07-17

**Authors:** Jesus Perez, Debra A Russo, Jan Stochl, Sarah Byford, Jorge Zimbron, Jonathan P Graffy, Michelle Painter, Tim J Croudace, Peter B Jones

**Affiliations:** 1 CAMEO Early Intervention Services, Cambridgeshire and Peterborough NHS Foundation Trust, Ida Darwin, Fulbourn, Block 7, Ida Darwin, Fulbourn, Cambridge, CB21 5EE, UK; 2 Department of Psychiatry, Herchel Smith Building for Brain and Mind Sciences, University of Cambridge, Forvie Site, Robinson Way, Cambridge, UK; 3 Centre for the Economics of Mental and Physical Health, Institute of Psychiatry, King's College London, 16 De Crespigny Park, London, SE5 8AF, UK; 4 Primary Care Unit, Department of Public Health and Primary Care, Institute of Public Health, University of Cambridge, Forvie Site, Robinson Way, Cambridge, CB2 0SR, UK; 5 Department of Health Sciences, Seebohm Rowntree Building, University of York, Heslington, York, YO10 5DD, UK; 6 National Institute of Health Research (NIHR) Collaboration for Leadership in Applied Health Research and Care for Cambridgeshire and Peterborough (CLAHRC-CP), 18 Trumpington Road, Cambridge, CB2 8AH, UK

**Keywords:** Early intervention, Primary care, Psychosis, Cluster randomized controlled trial, Ultra high-risk

## Abstract

**Background:**

The early detection and referral to specialized services of young people at ultra-high risk (UHR) for psychosis may reduce the duration of untreated psychosis and, therefore, improve prognosis. General practitioners (GPs) are usually the healthcare professionals contacted first on the help-seeking pathway of these individuals.

**Methods/Design:**

This is a cluster randomized controlled trial (cRCT) of primary care practices in Cambridgeshire and Peterborough, UK. Practices are randomly allocated into two groups in order to establish which is the most effective and cost-effective way to identify people at UHR for psychosis. One group will receive postal information about the local early intervention in psychosis service, including how to identify young people who may be in the early stages of a psychotic illness. The second group will receive the same information plus an additional, ongoing theory-based educational intervention with dedicated liaison practitioners to train clinical staff at each site. The primary outcome of this trial is count data over a 2-year period: the yield - number of UHR for psychosis referrals to a specialist early intervention in psychosis service - per primary care practice.

**Discussion:**

There is little guidance on the essential components of effective and cost-effective educational interventions in primary mental health care. Furthermore, no study has demonstrated an effect of a theory-based intervention to help GPs identify young people at UHR for psychosis. This study protocol is underpinned by a robust scientific rationale that intends to address these limitations.

**Trial registration:**

Current Controlled Trials ISRCTN70185866

## Background

The detection and prompt referral to early intervention services of young people who may be at ultra-high risk (UHR) for psychosis [1] is intended to reduce the duration of untreated psychosis (DUP) and improve outcomes [2]. Early referral is, therefore, a desirable behavior in professionals who have the opportunity to do so.

General practitioners (GPs; primary care physicians) are usually the healthcare professionals contacted first by individuals at UHR for psychosis [3]. However, early detection of psychosis in primary care is difficult because of the nonspecific nature of behavioral and psychological antecedents of psychosis, and the very low predictive value [4].

Some Scandinavian and Australian projects have developed protocols for the detection of people at UHR for psychosis in professional settings such as primary care [5,6]. However, none of them have evaluated the effectiveness or cost-effectiveness of different approaches. Such analyses may be important because education alone fails to improve the management and identification of mental health problems in primary care [7]. Indeed, a recent educational intervention that attempted to enhance GP skills in the identification of first-episode psychosis (FEP) neither modified referral rates to early intervention services nor reduced the DUP [8].

We present here the design and implementation of the first cluster randomized controlled trial (cRCT) that compares two different approaches to liaising with primary care, in order to increase detection of young people at UHR for psychosis and early referral to a specialist early intervention team. The approach and methodology follows the Medical Research Council (MRC), London, UK, guidelines for the design and evaluation of complex interventions [9].

## Methods/Design

### Cluster randomized controlled trial (cRCT)

#### Aim

To test the null hypothesis that a theory-based educational intervention for primary care, including ongoing personal liaison by specialist health professionals, is not different, in terms of effectiveness and cost-effectiveness, to a postal information campaign coordinated from an office in a specialist, secondary care-based, early intervention service (CAMEO, Cambridgeshire and Peterborough, UK; http://www.cameo.nhs.uk), for detecting individuals aged 16 to 35 years at UHR for psychosis in primary care.

In this cRCT, primary care practices across Cambridgeshire and Peterborough are allocated to one of the following educational groups and referral activity is followed over a period of 2 years:

1.) Low intensity: implementation of the postal information campaign that represents a minimum of good practice.

2.) High intensity: implementation of the postal information campaign plus an additional, ongoing theory-based educational intervention.

#### Identification and recruitment of practices

A total of 104 general practices, working across 138 surgeries (sites), within the geographical boundaries of Cambridgeshire and Peterborough, were identified from the Primary Care Research Network East of England (PCRN EoE; http://www.crncc.nihr.ac.uk/about_us/pcrn/eoe) database. All had practice nurses and varied from single-handed to multi-partner practices, with the largest practice having 15 GPs. They included a mixture of urban, suburban and rural practices.

The original design of the trial was predicated on the presumption that formal consent to take part in the study was not required because the study would not directly involve patients or their care, and was understood in the context of service development within the National Health Service (NHS) in primary care. Clinical equipoise was assumed and resource constraints precluded implementation in all practices. Thus, the trial would involve all practices across Cambridgeshire and Peterborough. However, the Cambridgeshire 1 Research Ethics Committee granted approval on the basis that consent was obtained from general practices to take part in the study, which represented a significant change in the design. Even if formal consent had not been required, the study team would still have needed the agreement of practices in the high intensity arm to undertake elements of the study, such as the educational sessions and distribution of leaflets among staff. The invitation to participate may therefore have influenced referral behavior in practices that did not consent to participate. We are, however, routinely collecting information regarding the number of UHR and FEP referrals from these sites as part of our ongoing clinical service evaluation. We will also analyze the characteristics of these practices in order to evaluate the validity of our findings.

Following the Ethics Committee’s requirements, the partners at each practice were provided with an information sheet (available from the authors) detailing the study. They were then contacted by the PCRN EoE and research team, and asked whether they were interested in taking part. If they expressed interest, a liaison practitioner (LP) visited to obtain a signed consent form from the partner. A GP or nurse at the surgery (site) was identified to act as the point of contact should they be randomized to the high intensity arm.

All the clinical staff from practices randomized to the high intensity arm will be invited to attend educational sessions organized at their respective surgeries, and their time reimbursed by the West Anglia Comprehensive Local Research Network (West Anglia CLRN; http://www.crncc.nihr.ac.uk/about_us/ccrn/west_anglia) as service time spent on the research.

#### Randomization of clusters

General practices were considered as the clusters and randomized at this level, since some practices operated from more than one surgery and shared clinical staff. Practices that consented to participate in the study were stratified according to three high-level factors that were considered, *a priori*, to be likely to relate to referral behavior:

1.) Three levels of geographical area: Cambridge and South Cambridgeshire, Huntingdon and East Cambridgeshire, and Peterborough and Fenland.

2.) GPs working at multiple sites (yes/no).

3.) Membership of the Association of Student Practices in Cambridge (N=8) where university students account for a high proportion (approximately 50%) of the total list size.

**Figure 1:**
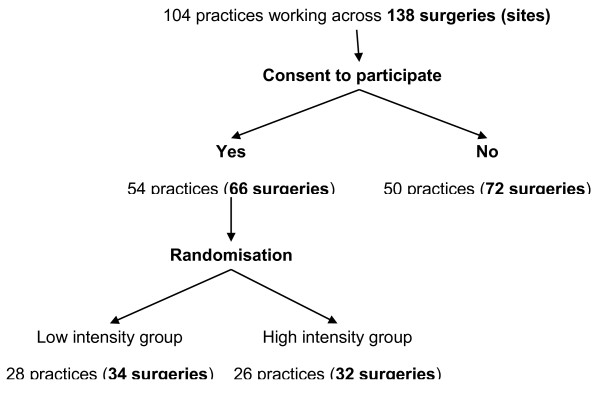
Flow of practices/surgeries through selection, consent and randomisation processes.

Randomized allocation was carried out independently of the research team and occurred after obtaining consent. Randomization was in 12 strata and 96 blocks, with block size 2 (‘ralloc’ command in Stata (StataCorp JP, College Station, TX, USA)) (Figure 1).

#### Primary outcome

The primary outcome of this cRCT is count data over a 2-year period: the yield - number of UHR for psychosis referrals to a specialist early intervention in psychosis service (CAMEO) - per practice site.

#### Secondary outcomes

New trial-initiated referrals will be assessed by the study team and stratified into individuals who meet criteria for UHR for psychosis or FEP according to the Comprehensive Assessment of At-Risk Mental States (CAARMS) questionnaire (true positives) [10], and people who do not fulfill the criteria (false positives).

We will also perform an economic evaluation that will comprise two components:

1.) Evaluation of the short-run cost-effectiveness of both educational strategies in terms of incremental cost per true positive detected.

2.) Evaluation of longer-term cost-effectiveness using decision analytic techniques.

Data on resource use for costing purposes will be recorded using the Adult Service Use Schedule (AD-SUS) modified for early intervention (EI-ADSUS). The EI-ADSUS was designed on the basis of previous economic evidence in relevant mental health populations [11,12] and was adapted for early intervention following consultation with the clinical team and evidence from the literature [13].

#### Sample size calculation

We powered the study based on sample size formulae for Poisson outcomes in a completely randomized design. For power of 80% with: 1) a significance level of 0.05 (two-sided); 2) referral counts expressed as an incidence rate of referrals in the low intensity group of 40 per 100,000 person-years [14]; 3) an anticipated incidence rate in the high intensity group of 0.00080 per 100,000 person-years; 4) 2,000 person-years per cluster (the average surgery list size for the age range of 16 to 35 years, per 2 years of study); and 5) a coefficient of variation estimated at a value of 0.15, our calculations required a sample size of 31 surgeries (practice sites) in each arm.

#### Statistical analysis

In the pre-modeling phase of our analysis, basic descriptive statistics for the total number of referrals, including proportion of true and false positives, will be provided. Subsequent analyses will be carried out separately for true and false positives.

Given that the outcomes are count data, our primary statistical method for modeling will be Poisson regression. If the assumptions of Poisson regression are not met (for example overdispersion), we will use zero-inflated or negative binomial regression models. The fit of the model to the data will be evaluated by comparison of model log-likelihoods. The results will be adjusted for surgery size, considering the number of GPs working in each site as a covariate in the model.

#### Economic analysis

The economic evaluation takes a broad public sector perspective, including the cost of all health and social services, criminal justice sector costs, and productivity losses as a result of time off work due to illness. The high intensity intervention will be costed on the basis of contact data from records and the salary of the liaison practitioners delivering it, including employer costs (National Insurance and superannuation contributions) and overheads (capital, administrative and managerial) [15]. Other unit costs will be taken from published sources [15-19]. Productivity losses, for young people who are working, will be calculated using the human capital approach, which involves multiplying time off work due to illness by the participant’s salary [20]. Analyses of total cost will compare mean costs using standard *t*-tests to enable inferences to be made about the arithmetic mean [21], and the validity of the results will be confirmed using bias-corrected, non-parametric bootstrapping [22].

Short-run cost-effectiveness will be assessed in terms of incremental cost per true positive and detected using the net benefit approach [23]. The incremental cost-effectiveness ratio (ICER) will be based on parameter estimates from bivariate random effects (‘multilevel’) models, which model costs and outcomes simultaneously, taking account of the hierarchical structure of the data in cluster randomized trials [24,25]. The parameters from the bivariate model will be used to construct cost-effectiveness acceptability curves (CEACs), a recommended decision-making approach, which describes uncertainty around the estimates of expected costs and effects. CEACs are presented by plotting the probability of the intervention being cost-effective for a range of possible values of willingness to pay for a unit improvement in outcome [26]. Since the short-term cost-effectiveness analysis focuses on identification of young people at risk, and not the outcomes for these individuals, decision analytic modeling will be used to explore the relative cost-effectiveness of the interventions in the longer-term [27]. Decision modeling allows assessment of the mean expected costs and outcomes for each arm of the study by modeling a hypothetical cohort of young people identified as at risk. The model will be populated using data on sensitivity and specificity from the cRCT, and data on longer-term care pathways, probabilities, costs and outcomes from a systematic review of the literature. Should gaps remain, expert opinion will be sought [28].

We will select the most suitable modeling framework in which to carry out the analysis, dependent upon the results of the cRCT and the availability of suitable data from the literature. In cases where individuals can be regarded as independent and interaction between them is not an issue in terms of the course or progression of an illness, as is the case in the current population, either a decision tree or a Markov model may be appropriate [29]. Model parameters will be entered into the model with associated probability distributions to explore uncertainty using Monte Carlo simulation, and probabilistic sensitivity analysis will be used to explore the robustness of the model and the impact of alternative model assumptions [27].

### Low intensity: the postal information campaign

The main element of the postal information campaign is a specifically designed laminated leaflet (available from the authors), which provides guidelines to help GPs identify and refer individuals at UHR for psychosis. This leaflet will be posted to the surgeries in the low intensity group and integrated within the high intensity educational program (‘high intensity intervention’) to allow investigation of the research question.

Another leaflet had been in routine use for some years in CAMEO, but the trial presented an opportunity to revise it, while not radically changing routine practice in our particular early intervention service that is similar to other services in the region. For the design of the new leaflet, we followed a joint initiative from the MRC and British Psychological Society (BPS), UK, to examine the scientific understanding of the psychological processes involved in the implementation of evidence-based practice guidelines in health services. They recommended the following: 1) guidelines are more closely followed if the wording of behavioral instruction is concrete and precise; 2) the more precisely behaviors are specified, the more likely they are to be carried out; and 3) specifying what, who, when, where and how will assist implementation [30].

In addition, the research team collaborated with a designated advisory group of professionals, including psychologists, psychiatrists, NHS Trust communication teams and GPs. The consensus was that the leaflet should be A4 portrait and on one side only, brief and informative, and with the capacity to be used as a tool for identifying symptoms of individuals at UHR for psychosis. The leaflet was laminated for durability. Amendments and additions were incorporated through several cycles of drafting until a final agreement was reached.

The fundamental requirements were:

1.) An outline of the rationale for early detection of individuals at UHR for psychosis. This included points that were pertinent to GPs (for example halves the risk of suicide), as it has been shown that the more relevant or important the information is to the reader, the more likely they will spend time processing the information [31-34].

2.) A brief description of how individuals at UHR for psychosis might present and who to refer.

3.) Examples of questions for GPs to ask potential individuals at UHR for psychosis to help elicit vital information about underlying sub-threshold psychotic symptoms. This was designed to address Lester *et al*.’s (2005) important finding that GPs had concerns regarding how to phrase sensitive questions about hallucinations, thought disorder and suicide [35].

4.) A list of criteria that would indicate a referral. These were based upon the symptoms included in the CAARMS [10]. Items were presented in a tick-box format because it is well-documented that passive presenting of information is less effective than prompting individuals to engage with the material [36]. Additionally, the use of an algorithmic format has contributed to successful guideline use [37]. Special attention was paid to ensure that the criteria achieved a balance between sensitivity and specificity.

5.) Prominent contact details to facilitate referrals.

### Design of the high intensity intervention

The MRC framework (2008) for the development and evaluation of a complex intervention was used to guide the development of the educational intervention [9]. We also referred to a review providing guidance on the essential components of an effective educational intervention in primary mental health care [32]. This recommended that learning components of the intervention should demonstrate a clinical need and facilitate practical application of new knowledge using examples and data from personal clinical practice. This is essential if clinicians are to recognize their potential needs for improvement. Also, interventions should be multifaceted and supported by practice-based contacts for a period of follow-up.

In conjunction with the factors outlined, we addressed the absence of an explicit, theoretical framework in the design of many educational interventions to change professional practice [38]. We considered the purpose of the intervention to be a change in behavior on the part of professionals.

#### Theoretical framework

There is growing evidence to support the application of psychological models of behavioral change to the clinical behavior of healthcare professionals [39,40]. It helps identify mediators of clinical decision-making [41], and thus allows appreciation of the causal mechanisms responsible for any observed behavior change and valid conclusions concerning the efficacy of the intervention [42].

The theory of planned behaviour (TPB) [43,44] was selected to underpin this research. The TPB is increasingly used to predict intentions and behavior in relation to clinical practice [45]. Ramsey *et al*. (2010) concluded that the TPB was an appropriate theory to predict healthcare professional behavior change and that it offered insight into the processes underlying change in educational interventions in primary care [46]. More pertinent to mental health issues, Green *et al*. (2008) found TPB predictors explained 86% of the variance in GPs’ intentions to refer patients to specialist eating disorder services [47].

The TPB would propose that the act of identifying individuals at UHR for psychosis in primary care is predicted by the strength of a GP’s intention to identify these individuals. This intention is influenced by three predictor variables: 1) whether the GP is in favor of identification (attitude); 2) the intensity of social pressure the GP perceives (subjective norm); and 3) how much the GP feels in control of this identification (perceived behavioral control (PBC)). The measurement of these predictors, pre- and post-intervention, and analysis of their relationship with our objective outcome measure (number of referrals), will enable evaluation of the effect of the intervention on actual behavior and the underlying behavioral process that drive it.

#### Feasibility of theory of planned behaviour (TPB) in primary care

Use of the TBP to design interventions requires the development of a questionnaire to allow the identification and measurement of specific beliefs associated with each construct (intention, attitude, subjective norm and PBC). These beliefs can then be targeted with strategies designed to influence these constructs in the appropriate direction. Strengthening practitioner intentions can be expected to change practitioner behavior in identifying individuals at risk.

In accordance with the TPB guidelines [48,49], pilot work was undertaken away from the study area to identify accessible behavioral, normative and control beliefs. This confirmed the feasibility, reliability and acceptability of administering a TPB-based questionnaire within a representative sample of GPs, to identify beliefs and intentions concerning the identification of individuals at UHR for psychosis. This development and the resulting questionnaire used in this trial are described in detail, elsewhere [50].

#### What TPB predictor variables will be targeted?

A crucial factor in the decision-making process of TPB variables to target was the experimental design of the trial. Strong internal validity is necessary to determine whether the intervention program affects the main outcome measure. If discrete cluster questionnaire scores for the TBP variables are taken into account and predictor variables are targeted accordingly, surgeries in the high intensity group would receive differing interventions. It would then be impossible to make comparisons with the low intensity clusters, draw conclusions about the efficacy of the intervention and identify the potential causal mechanisms for any observed change. Furthermore, research has confirmed that simultaneous maximization of all three TPB variables generated the largest increase in intentions [51]. Therefore, it was decided that all three TPB predictor variables would be targeted for each surgery.

#### Selection of behavior change techniques for TPB predictor variables

We employed the TPB coding manual developed by Abraham and Michie (2008) [52] to match the three TPB predictor variables to the theoretical construct domains. We then used a tool developed by Michie *et al.* (2008) [53], which associates these theoretical constructs with the most effective behavior change techniques (Table 1).

**Table 1:** Behavior change techniques to facilitate theory of planned behavior (TPB) constructs throughout the trial

**TPB construct**	**Behavior change technique**	**Procedures and materials**	**Delivery context**
**Attitude**	Provide general information on behavior-benefit link	Leaflet: distributed by post, one for each GP in each surgery. Outline the benefits of the early detection of psychosis.	Pre-sessions 1 and 2
		PowerPoint presentation: provide information about physical, psychological and social benefits of identifying potential individuals at UHR for psychosis.	Sessions 1 and 2
		DVD: the above points are reiterated by the head of the department of psychiatry, a well-respected authority in the trial’s area.	Session 2
	Provide information on consequences	PowerPoint presentation: provide information on the consequences of employing a ‘wait and see’ strategy with potential individuals at UHR for psychosis; reducing involvement with police and/or hospital admissions that often occur prior to a FEP.	Sessions 1 and 2
		DVD: include a vignette showing the possible consequences of a GP employing a ‘wait and see’ strategy with a individual at UHR for psychosis.	Session 2
	Provide information about personal susceptibility to negative consequences	PowerPoint presentation: provide peer-reviewed research evidence showing the importance of GPs in the care pathway of individuals at UHR for psychosis; linking with the potential costs of inaction by the GP.	Sessions 1 and 2
	Provide information about severity of health consequences	Leaflet: outline the potential to reduce suicide attempts.	Pre-sessions 1 and 2
		PowerPoint presentation: outline the link between delayed detection and transition to FEP; provide research data showing the poor outcomes for individuals who transition.	Sessions 1 and 2
		DVD: the above points are reiterated by the head of the department of psychiatry.	Session 2
**Subjective norm**	Provide information about others’ approval	Produce newsletter for dissemination to each GP in all surgeries via post and email.	3 × yearly throughout the trial
		Include details of the number of surgeries participating and positive quotes from GPs about the consequences of participating in the trial.	
	Provide normative information about others’ behavior	Produce newsletter for dissemination to each GP in all surgeries via post and email.	3 × yearly throughout the trial
		Include information about the number of surgeries participating in the trial.	
		Provide an update of the number of referrals in the trial, and true UHR and FEP cases in the county.	
	Prompt identification as role model/position advocate	Identify a LEGS ‘champion’ within each surgery to promote the identification of individuals at UHR for psychosis and raise any issues or problems at weekly meetings.	Post-session 1
	Provide opportunities for social comparison	Opportunities for peer interactions are facilitated by the group setting, and encouraged by LPs concerning potential advantages and facilitators of the identification of individuals at UHR for psychosis.	Sessions 1 and 2
		Opportunities for peer interactions are facilitated by the group setting and encouraged by LPs concerning previous referrals, sharing experience and discussing helpful strategies.	Session 2
		Group discussions and LPs reinforce social approval of the identification of individuals at UHR for psychosis.	Sessions 1 and 2
**Perceived behavioral control (PBC)**	Prompt barrier identification	Barrier identification based on responses to the PBC items within the TPB questionnaire.	Sessions 1 and 2
		Group discussions of possible barriers and means to minimize or address them.	Sessions 1 and 2
		Provision of strategies to overcome barriers, for example educate GPs to ask the most relevant questions to identify UHR for psychosis; therefore, making optimal use of the limited consultation time.	Sessions 1 and 2; and throughout the trial when appropriate during telephone and face-to-face contact with GPs

	Provide general encouragement	LPs to provide general encouragement on a one-to-one basis, as and when needed, and during the educational sessions to the surgery as a whole.	Throughout the trial during telephone and face-to-face contact with GPs
	Provide instruction	PowerPoint presentation: instruction on the appropriate questions to ask potential individuals at UHR for psychosis; how to refer, care pathway slide.	Session 1
		Leaflet: include examples of the questions to ask patients and tick-box options of the appropriate criteria required for a referral.	Pre-sessions 1 and 2
		DVD: outline in more detail the early signs and symptoms to be aware of, examples of questions, and how to refer using a question and answer format, with a GP and the head of the department of psychiatry.	Session 2
	Model/demonstrate the behavior	DVD: instructional vignettes showing examples of a GP conducting a consultation with a potential individual at UHR for psychosis, before and after the educational sessions.	Session 2
	Provide feedback on performance	Provided for each GP for every referral, both verbally and in a letter; include detailed feedback on the outcome of the initial assessment to explain why, or why not, the individual met the criteria for UHR for psychosis.	Throughout the trial
		PowerPoint presentation: feedback table for the previous year’s referrals associated with each surgery, including source, outcome and any signposting to other services. Facilitate discussion around the reasons why they did, or did not, meet criteria.	Session 2
	Prompt practice	LPs to prompt practice on a one-to-one basis, as and when needed, and during the educational sessions to the surgery as a whole.	Throughout the trial during telephone and face-to-face contact with GPs
	Use of follow-up prompts	Leaflet: use as a reminder to prompt practice.	Pre-sessions 1 and 2
		Newsletter: use as a reminder to prompt practice.	3 × yearly throughout the trial
	Time management	Leaflet: strategy for optimal use of the limited consultation time.	Pre-sessions 1 and 2
		PowerPoint presentation: strategy for optimal use of the limited consultation time.	Session 1
		DVD: strategy for optimal use of the limited consultation time.	Session 2
	Prompting focus on past success	PowerPoint presentation: feedback table for the previous year’s referrals associated with each surgery, prompting focus on the appropriate referrals to increase PBC.	Session 2; and when appropriate during telephone and face-to-face contact with GPs
	Provision of general information	General introduction to rationale and aims of the trial.	Sessions 1 and 2
		General introduction to UHR for psychosis definitions and concepts.	
		Information about the early detection-improved outcomes link.	
		Illustrate the parallels between the trial’s aims and NICE recommendations for early intervention.	
**Intention**	Prompt general goal-setting and behavioral resolution	Encourage use of leaflet: prompt GPs to develop strategies to help remind them to use the leaflet for potential individuals at UHR for psychosis.	Sessions 1 and 2; and throughout the program, every time contact is made with the GP
	Prompt review of behavioral goals	GPs are asked to review a list of possible goals or plans they may have used to prompt or instigate the process of identification of individuals at UHR for psychosis, and indicate which strategies they used in the last year and which strategies would be useful in the following year.	Included within TPB questionnaire in session 2; and a copy provided for each GP for future reference

Based on Michie *et al*. (2008) [53]. *FEP* first-episode psychosis, *GP* general practitioner, *LEGS* liaison with education and general practices, *LP* liaison practitioner, *NICE* National Institute for Health and Care Excellence, *PBC* perceived behavioral control, *TPB* theory of planned behaviour, *UHR* ultra-high risk.

### Implementation of the high intensity intervention

#### Liaison practitioners (LPs)

Three dedicated LPs were specifically recruited for the trial to deliver the intervention (one male, two female; mean age 45.5 years, SD 4.7). All are experienced mental health professionals. Their principal function is to act as facilitator, since it is proposed that this is a fundamental role in helping individuals and teams to understand what they need to change, and how they need to change it, in order to translate evidence into practice [54]. Each LP is responsible for delivering the intervention to the surgeries within one of the three previously mentioned geographical boundaries in Cambridgeshire and Peterborough, regardless of the other two strata.

#### Components of the intervention

The educational components were designed to be multi-faceted and combine different means of dissemination (for example DVD, PowerPoint (Microsoft, Redmond, WA, USA) presentation, paper-based printed material and outreach visits) as this strategy has been shown to be the most effective when attempting to change clinician behavior [32,34,55-57]. All the educational materials have a clear visual identity and incorporate a specifically designed trial logo, with a recognizable combination of colors that reflect CAMEO branding. This mainly attempts to create a connection between the trial and the GPs, and an association that prompts GPs to think about identifying individuals at UHR for psychosis.

According to the TPB, attitude, subjective norm and PBC towards identifying individuals at UHR for psychosis cannot be directly manipulated; changes in these predictor variables are assumed to follow from changes in salient beliefs associated with the target behavior [58,59]. Therefore, the behavioral, normative and control beliefs generated from the pilot study [50] guided the development of the materials and strategies included in the intervention. Lack of awareness, familiarity and agreement with the UHR for psychosis concept, self-efficacy and outcome expectancy, in addition to external barriers, and GPs’ perceptions of what colleagues and significant others expected of them, will be targeted with theory-driven strategies throughout the intervention. The aim is to encourage GPs to identify individuals at UHR for psychosis by incorporating apposite knowledge and skills into their practice.

#### Duration of the intervention

The intervention will be implemented over a period of 2 years, since enhanced outcomes have been obtained with interventions that repeat activities and reminders at intermittent intervals [32]. Furthermore, previous research suggests that clinicians do not adopt research findings directly, but need time to process, assimilate and apply the information to their own needs and practice [60].

#### Educational sessions

Practice-based educational sessions were chosen since it has been suggested that outreach visits may be the most effective strategy in the introduction of new clinical guidelines and influencing professional behavior [32,57]. This would also allow comparisons of cost-effectiveness between a resource-intensive strategy and a simple postal campaign.

An initial 1-hour educational session on UHR for psychosis detection will be followed 1 year later by a booster 1-hour session to: reiterate the main messages; consolidate skills and knowledge; discuss particular practical scenarios which could emerge during the course of the study; and adjust or improve ongoing intensive liaison techniques if required. All three TPB predictor variables (that is attitude, subjective norms and PBC) and intention will be targeted in both educational sessions. Accordingly, the components of the first educational sessions will be:

1.) TPB questionnaire

the TPB questionnaire [52] will provide a measure of the proposed mechanisms that mediate GP’s behaviour.

2.) PowerPoint presentation

the research team collaborated with the designated advisory group to generate and agree the content, format and layout of the presentation. A script was produced to ensure all LPs delivered the same content to each surgery. The presentation will cover the following items: a) the trial; b) the benefits of early detection for psychosis; c) the role of GPs in the trial; d) presentation of individuals at UHR for psychosis in general practice; e) referral procedure to CAMEO; and f) time to raise questions and discuss potential problems.

3.) Pack

while passive dissemination of printed educational materials alone have little effect on changing clinician behavior [32,34,61], Wensing and Grol (2005) proposed that they can reinforce outreach educational strategies by addressing barriers to change, and consequently facilitate modifications in clinicians’ behavior [56]. Therefore, an information pack will be provided for each GP, including handouts of presentation notes, a reference list, paper copies of the trial leaflet, local early intervention services leaflet, a copy of the trial information sheet and contact details of the surgery’s designated LP.

The second educational session will include:

1.) TPB questionnaire

it will contain an additional item in the questionnaire to help target the TPB variable intention. GPs will be asked to indicate which strategies they used in the last year and which strategies would be useful in the following year.

2.) PowerPoint presentation

the second presentation will include a brief recap of salient points covered in first session, feedback and a review of the practice’s referral history to CAMEO since the trial began. As Howe *et al*. (2006) identified that successful educational interventions conducted in primary mental health care used personalized material and data based on the clinicians’ own performance and/or patients [32], LPs will prompt discussion to actively involve GPs in an examination of their referral history, and consider problems and implications for their clinical practice.

3.) DVD

a video was well-received in an educational intervention to improve detection of FEP in primary care [35]. Therefore, an educational DVD was produced for the present trial, incorporating some of their ideas and techniques, together with novel approaches more relevant to our target population and topic. In conjunction with a broadcast media developer from the Media and Systems Group within the Anglia Support Partnership, Huntingdon, UK, and the designated advisory group, the research team developed an 18-minute DVD.

Considering that vicarious experience of a required behavior has been shown to increase self-efficacy [62,63], observing another GP implementing successful UHR for psychosis identification can demonstrate that it is achievable and might motivate GPs to attempt the same. The educational DVD was designed to provide this experience to GPs in the trial by depicting GP consultations with potential individuals at UHR for psychosis.

Opinion leaders can be persuasive agents of behavioral change [64]. These individuals are defined as credible individuals within a particular social and professional network who have significant influence over others [65]. Thus, Professor Peter B Jones (PBJ), Head of the Department of Psychiatry, University of Cambridge, Cambridge, UK, presented the DVD. Actors were used to portray the roles of the GP (an occupational therapist) and patient (a research facilitator from the East Anglia Hub of the Mental Health Research Network (MHRN)). The DVD was filmed in and around a local general practice surgery for authenticity.

The content of the DVD included an introduction by PBJ explaining the concept of UHR for psychosis and emphasized the importance of early detection to improve outcomes, outlining the key role of GPs.

Two consultation scenarios were used to reinforce the need to lower clinical threshold and consider the possibility of sub-threshold psychotic symptoms underlying precursors such as reduced functioning, poor sleep etc. The first scenario depicts a young person experiencing negative thoughts and perceiving individuals laughing at her and calling her derogatory names, but presenting to the GP with concentration and sleep problems which result in difficulties keeping up with college work. The GP employs a “watch & wait” strategy by asking the patient to return in several weeks. The second scenario depicts the same patient with the same symptoms. This time, the GP asks the supplementary questions provided on the leaflet and emphasised in the educational content. UHR symptoms are elicited and a referral is made to CAMEO for further assessment.

The scenarios were interspersed with short discussion segments by PBJ to emphasize and reiterate the salient points, and a question and answer session between the GP and PBJ to address prevalent beliefs elicited in the pilot questionnaire [50], for example why should a ‘wait and see’ strategy be avoided?

4.) Pack

in addition to the relevant information for this second session, a copy of the above mentioned DVD will be included.

#### Written feedback for every referral

In order to provide personalized feedback, GPs in the high intensity group will receive a more detailed written feedback for every assessment throughout the trial period. A template was designed to ensure consistency and accuracy. This described the outcome of the CAARMS and why, or why not, the patient did, or did not, meet the criteria for UHR for psychosis.

#### Ongoing support

Repeated contact and reminders appear to be more important in provoking a change in GP behavior than total time input [32,34]. Indeed, the effect of outreach can double with just one repeat contact [61]. In order to continue the intervention between and after the two sessions, every practice will be offered support and training in the form and frequency that best suits their particular needs, based on the information collected from the sessions.

#### Newsletter

Results from the pilot study [50] revealed that intentions to identify individuals at UHR for psychosis were most strongly predicted by subjective norms. This implies GPs’ perceptions of whether other GPs identify individuals at risk, and whether colleagues or the healthcare system approve, or disapprove, of UHR for psychosis identification, are prominent motivational factors. Therefore, a regular newsletter reporting the participation rates and referral rates for the whole trial area was chosen as a strategy to target this potential causal mechanism to prompt behavioral change.

The thrice yearly newsletter will include graphs to present: 1) the number of referrals that met the criteria for FEP and UHR for psychosis for each of the geographical areas within the county; and 2) a comparison of the number of referrals from primary care and secondary care. This will demonstrate to GPs that other surgeries may also be referring individuals at UHR for psychosis to the trial, while raising awareness that there may be individuals at UHR for psychosis reaching secondary care services before referral to CAMEO.

#### Feedback questionnaire

To enable assessment of the acceptability and perceived effectiveness of the first year’s intervention on GP learning, a feedback questionnaire will be sent to each GP 1 month before the second session is due. This information will also be used to tailor the second year of the intervention to include strategies that focus on the needs of individual surgeries, because the closer educational material is connected to real problems, the greater the application of new learning [32,33].

#### Completion of the intervention

The main outcome measure will enable assessment of changes in behavior and allow conclusions to be drawn concerning the efficacy of the educational intervention. However, supplementary evaluation information is beneficial to summarize the spectrum of knowledge, skills and attitudes learned, and also appreciate the suitability and appropriateness (feasibility and acceptability) of replicating the intervention in other settings [46]. To obtain this information, GPs will be asked to complete a second, more comprehensive feedback questionnaire at the end of the intervention. This will contain items to evaluate each of the intervention components and assess relevance of the intervention, in addition to satisfaction and enjoyment.

## Discussion

There is little definitive guidance on the essential components of an effective educational intervention in primary mental health care. Lester *et al.* (2009) designed an educational intervention for GPs addressing knowledge, skills and attitude about FEPs [8]. Results indicated that training is insufficient to alter referral rates to early intervention services or reduce the DUP. This is not an unusual phenomenon; many well designed studies with demanding training interventions in primary care mental health failed to show significant outcomes [32].

Power *et al*. (2007) reported results for an intervention comprising GP education and direct access to an early detection assessment team [66]. In contrast to Lester *et al*. (2009) [8], this intervention significantly increased referral of patients directly to mental health services; fewer patients experienced long delays in receiving treatment, compared with the control group receiving standard local mental health services without the addition of GP training [66]. Most recently, Simon *et al*. (2010) used a sensitization model to increase GPs’ awareness of the warning signs in prodromal schizophrenia; three sets of clinical vignettes were sent by post to a randomly selected group of GPs in Switzerland. Results showed that sensitized GPs demonstrated a significant increase in diagnostic knowledge at 6 and 12 months, compared with both baseline knowledge scores and to GPs who were not sent the materials [67]. However, this study did not assess whether an increase in diagnostic knowledge resulted in a change in behavior in terms of more accurate or increased referrals to secondary care services.

Furthermore, the lack of an explicit theoretical framework in the designs of these three studies precluded appreciation of the causal mechanisms responsible for the observed improvement and valid conclusions concerning the efficacy of the intervention. Darker *et al.* (2010) claimed that the TPB has rarely been used to develop, design and evaluate interventions [68].

To our knowledge, no study has demonstrated an effect of a TPB-based intervention to help GPs identify people at UHR for psychosis on objectively measured behavior or examined whether the TPB constructs mediate the effects of an intervention on this behavior.

This cRCT attempts to address these limitations, ensuring that the intervention is underpinned by a robust scientific rationale which enables explanation of how and why each component of the intervention has any effect. This theoretical framework will also guide the process for evaluation and refinement of the intervention.

## Status of the trial

The trial has begun and general practices have been randomly allocated to the high or low intensity arms.

## Abbreviations

BPS: British psychological society; CAARMS: Comprehensive assessment of at-risk-mental-states; cRCT: Cluster randomised controlled trial; DUP: Duration of untreated psychosis; GP: General practitioner; LP: Liaison practitioner; MHRN: Mental health research network; MRC: Medical research council; PCRN EoE: Primary care research network east of England; TPB: Theory of planned behaviour; UHR: Ultra-high risk for psychosis.

## Competing interests

The authors have no conflicts of interest based on business relationships of their own or of immediate family members.

## Authors’ contributions

PBJ is the chief investigator for this trial. JP and MP are principal investigator and project manager, respectively. All authors participated in the design of the study. DAR elaborated the theoretical basis of the project. JS and TJC were in charge of sample size calculations, statistical analysis and randomization. SB developed the EI-ADSUS questionnaire for economic evaluation. JZ participated in the design of the postal information campaign. JPG provided valuable advice with regards to implementation and support required in primary care. JP and DAR drafted the manuscript. All authors provided a critical review and approved the final manuscript.
